# A Unified Conformational Selection and Induced Fit Approach to Protein-Peptide Docking

**DOI:** 10.1371/journal.pone.0058769

**Published:** 2013-03-13

**Authors:** Mikael Trellet, Adrien S. J. Melquiond, Alexandre M. J. J. Bonvin

**Affiliations:** Computational Structural Biology Group, Bijvoet Center for Biomolecular Research, Faculty of Science - Chemistry, Utrecht University, Utrecht, The Netherlands; Koç University, Turkey

## Abstract

Protein-peptide interactions are vital for the cell. They mediate, inhibit or serve as structural components in nearly 40% of all macromolecular interactions, and are often associated with diseases, making them interesting leads for protein drug design. In recent years, large-scale technologies have enabled exhaustive studies on the peptide recognition preferences for a number of peptide-binding domain families. Yet, the paucity of data regarding their molecular binding mechanisms together with their inherent flexibility makes the structural prediction of protein-peptide interactions very challenging. This leaves flexible docking as one of the few amenable computational techniques to model these complexes. We present here an ensemble, flexible protein-peptide docking protocol that combines conformational selection and induced fit mechanisms. Starting from an ensemble of three peptide conformations (extended, a-helix, polyproline-II), flexible docking with HADDOCK generates 79.4% of high quality models for bound/unbound and 69.4% for unbound/unbound docking when tested against the largest protein-peptide complexes benchmark dataset available to date. Conformational selection at the rigid-body docking stage successfully recovers the most relevant conformation for a given protein-peptide complex and the subsequent flexible refinement further improves the interface by up to 4.5 Å interface RMSD. Cluster-based scoring of the models results in a selection of near-native solutions in the top three for ∼75% of the successfully predicted cases. This unified conformational selection and induced fit approach to protein-peptide docking should open the route to the modeling of challenging systems such as disorder-order transitions taking place upon binding, significantly expanding the applicability limit of biomolecular interaction modeling by docking.

## Introduction

Among the wealth of protein-protein interactions that decide of the cell’s fate, peptides play a crucial role and account for about 40% of them [1]. From co-activators to inhibitors, they are involved in many signaling and regulation pathways and have been identified to interact with a large number of protein domains. MHC, SH3 and PDZ domains are for instance well known for their affinity toward peptide binding [2]–[4]. This large diversity of functions and the importance of the many biological pathways they mediate make them prone to be associated with diseases [5]. An emergent field in drug design focuses on the development of peptides for therapeutic applications [6]. Peptides have advantages over small-molecule inhibitors in that they can mimic protein-binding domains and are large enough to competitively inhibit protein–protein interactions. Pharmaceutical leads include for example antimicrobial peptides [7], [8], cyclic peptides [9] and also beta-breaking peptides that can inhibit amyloid fibril formation [10]–[12]. Another promising application field is that of fusogenic peptides used as cargo to deliver drugs to target cells [13].

Despite the large amount of data scientists have gathered over protein-peptide interactions [14], [15], structural determination of their complexes remains challenging due to two major obstacles: peptides are highly flexible and they often interact weakly with their substrate, underlining their importance in signal transduction or regulation which often relies on transient processes. These obstacles make experimental structure determination often non-trivial and call for complementary computational approaches like biomolecular docking. From a modeling perspective, conventional algorithms implemented either for protein-ligand or protein-protein docking are also often struggling with the problem of flexibility [16].

Few methods have been published to date to model peptides onto their protein receptors. Initial applications focused on specific protein families or domains involved in peptide recognition [2], [17]–[20], or were restricted to very short peptides [21]. Molecular dynamics simulations have also been used to predict protein-peptide interactions [22] but, even if providing interesting insights about the association process, they were only benchmarked against small sets of complexes and their applicability for the systematic screening of protein-peptide interactions remains to be demonstrated. FlexPepDock [23] was the first generic algorithm aiming at modeling near-native protein-peptide complexes, starting either from an ensemble of perturbed peptide structures or, with significantly less successful results, from an extended backbone conformation. FlexPepDock, which is also available as a webserver, assumes knowledge of the binding site (anchor residues) to build and refine the peptide onto its receptor [24]. When no information about the peptide backbone conformation is available, the same authors have proposed a much more computationally demanding pipeline [25] that combines Rosetta *ab-initio* predictions to ‘fold’ the peptide and FlexPepDock to refine the binding mode. Our own information-driven flexible docking approach HADDOCK [26], [27] has also been used in the past to model protein-peptide interactions, e.g. [28]–[32]. In HADDOCK, the docking is driven by (experimental) knowledge in the form of information about the interface region between the molecular components and/or their relative orientations, with applicability in protein-ligand, protein-nucleic acid and protein-protein docking predictions. HADDOCK can further handle simultaneously up to six molecules of various natures [33]. This integrative approach has proven its success in the blind international experiment CAPRI (Critical Assessment of PRedicted Interactions) [34] where it stands as one of the top-performing methods [35]. HADDOCK is also available as a web server [36], facilitating its usage for large community. Despite its successful application to the prediction of various protein-peptide complexes, HADDOCK’s performance for the modeling of protein-peptide interactions had not yet been systematically benchmarked nor had its protocol been thoroughly optimized for this purpose.

Development of a successful method for the modeling of protein-peptide interactions should also consider aspects of their molecular recognition mechanism. Over the last century several theories have been put forward to explain the molecular recognition process [37]–[41], among which induced fit [37], [38] and conformational selection [39]–[41]. Conformational selection postulates that, already in the absence of its ligand, a protein exists in a number of discrete conformational states in equilibrium, including the one that preferentially binds the ligand. This concept is opposed to the induced fit theory, primarily introduced to describe enzyme action, which states that the conformational fit is induced by substrate binding. In recent years, a shift toward a reconciliation of both models can be observed where conformational selection and induced fit may be in fact co-existing [42]–[45].

Here we present an optimized HADDOCK protocol for flexible protein-peptide docking that combines conformational selection and induced fit recognition mechanisms. The performance of this approach is demonstrated on a non-redundant protein-peptide benchmark, the peptiDB dataset [46]. The latter consists of both naturally occurring peptides and short segment of proteins, mainly loops or disordered regions that fold upon binding. It was originally developed to test the FlexPepDock algorithm [23]. We demonstrate that, using a coarse definition of the interacting surface on the unbound protein receptor and no information on the peptide side, HADDOCK is able to generate near-native or sub-angstrom models for ∼70% of the dataset in unbound/unbound docking.

## Results

**Figure 1:**
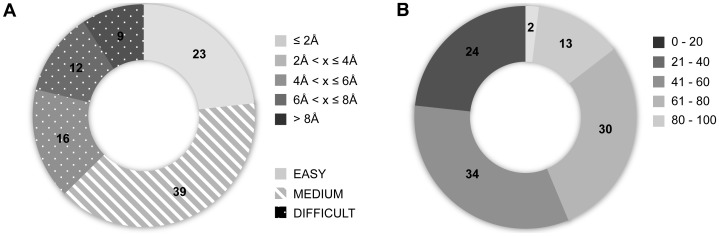
Protein-peptide benchmark characteristics. (**A**) Distribution of positional backbone RMSDs between the bound form of the peptides present in the benchmark and an ideal extended conformation. These are classified into three categories (easy, medium and difficult) based on the amplitude of the conformational change upon binding. (**B**) Percentage of solvent accessible residues computed for all peptides in the crystal structures of the respective protein-peptide complexes.

We have developed an efficient protein-peptide docking protocol that combines conformational selection with induced fit, capitalizing on two of HADDOCK’s features: *i)* its ability to provide ensembles of structures as starting point for the docking, and *ii)* its flexible refinement capabilities allowing for both backbone and side-chain flexibility. This protocol was optimized making use of the PeptiDB protein-peptide benchmark dataset [46] consisting of 103 non-redundant complexes, 62 of which also have the unbound form of the protein available. In the protein-protein docking field, the difficulty of a target is usually assessed by measuring the deviation between the unbound and bound forms of its constituents [34]. However, this measure is inapplicable within the context of protein-peptide docking since we usually don’t have access to the free form of the peptide. Therefore, in order to define the difficulty of each target, we measured instead the deviation in terms of backbone RMSD of the bound form of the peptide from an ideal extended conformation (see Methods). Three different classes were defined (easy/medium/difficult) (**Figure 1A**). The conformational changes on the protein side are, in first instance, not taken into account in this classification as most peptides were observed not to induce any significant conformational changes on their partner upon binding [46].

Since HADDOCK is an information-driven docking approach, in order to drive the docking, we defined a large binding site on the protein receptor derived from the interacting residues within 5 Å from the peptide in the crystal structures of the complexes (see Methods). On average, this maps a surface of 1200 Å^2^ on the protein side, whereas the average interface area in protein-peptide complexes is only about 500 Å^2^ (see **Table A in File S1**). This rather broad definition of the binding region was chosen to drive the docking to an approximate location of the peptide binding site without introducing too much bias in our results by defining the tight pocket or groove that accommodates the peptide. While this represents of course a “best case” scenario where the binding regions are rather well defined, this allows to concentrate on the problem of addressing properly the peptide flexibility in the docking process.

### Bound/Unbound (Extended) Docking – Impact and limitations of Flexible Refinement

**Figure 2:**
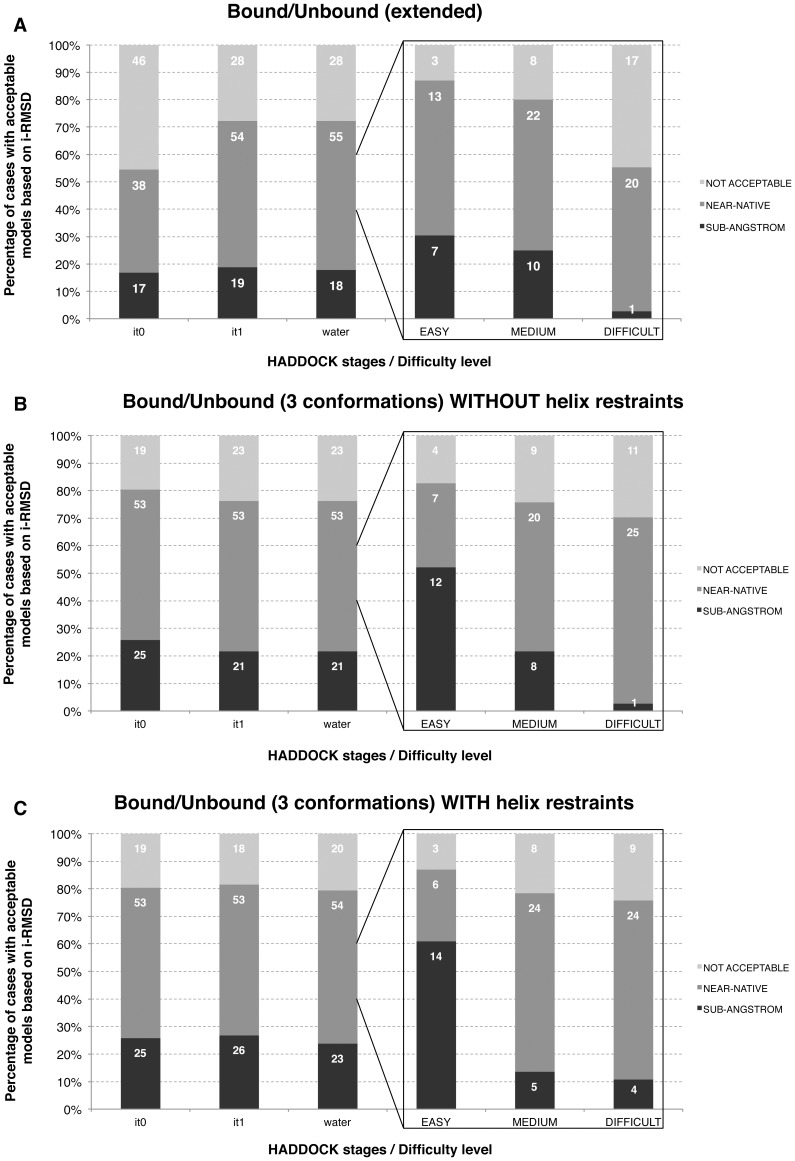
Overall HADDOCK results for (A) bound/unbound (extended), (B) bound/unbound (3 conformations) and (C) bound/unbound (3 conformations) with enhanced flexibility. The percentages of near-native and sub-angstrom resolution models at the various stages (rigid-body (it0), semi-flexible (it1) and water refinement (water)) are reported in the left panels and were calculated over the 400 final models generated by HADDOCK. The right panels show the percentages after water refinement as a function of the docking difficulty.

We first evaluated the performance of HADDOCK in docking and refining extended peptides. Based on the solvent accessible surface areas of the peptide in the crystal structures of the complexes (**Figure 1B**), in half of the cases the peptide targets a hollow surface on the protein and in another half, the peptide remains largely exposed to the solvent. Especially for the hollow binding sites, flexible refinement is crucial in generating proper poses. Rigid body docking only (it0 stage of HADDOCK) shows a success rate of 54% of acceptable (sub-angstrom or near-native) solutions (**Figure 2A**); while a subsequent refinement with both side-chain and backbone flexibility enables an induced fit of the peptides in their binding pocket, resulting in an 18% increase in performance leading to 72% of acceptable solutions over the benchmark. However, while peptides that bind onto their protein receptor in a stretched conformation (typically SH3 domains) can be successfully predicted, about 60% of the peptides binding in a helical conformation fail, which represents 44% of the failed cases (12 out of 28). Clearly, folding of a helix upon binding is far beyond the possibilities of the flexible refinement in HADDOCK. Other failed cases consists of deeply buried peptides and peptides with more complex and localized conformational changes including β-hairpins and turn-like conformations. The impact of the flexible refinement in terms of fraction of native contacts (Fnat) and RMSD improvements will be discussed later below.

### Bound/Unbound Docking – Introducing Conformational Selection

Given the failure to model helical peptide conformations starting from extended stretches, we revised our approach to allow for conformational selection at the rigid-body docking stage of HADDOCK. Among 103 complexes, 21 peptides bind onto their receptor in a helical conformation, 42 as extended or beta and the remaining 38 as disordered. This proportion reflects what other studies reported about the conformations adopted by peptides upon binding [1]. Building onto the ensemble docking capability of HADDOCK we started the docking from an ensemble of three distinct conformations of the peptide: α-helix, polyproline II and extended. Together, these three conformations cover about 80% of the observed peptide bound structures in the Protein Data Bank [47]. This combined conformational selection and induced fit protocol led to an increased success rate of 76.3% among the final models after final water refinement (**Figure 2B**). Not only does the performance improves, but also the quality of the generated models with 23 sub-angstrom high quality solutions compared to 17 for the induced fit approach only (**Figure 2B**).

In the cases where a helical conformation was selected, we however observed for some targets distortion of the helical conformation after flexible refinement. To correct this, we introduced “on-the-fly” backbone dihedral angle restraints for helical regions by allowing angle variations around the measured dihedral angle of ±10°. These restraints corrected the loss of secondary structure, improving substantially the overall success rate to 79.4% (**Figure 2C**). More specifically, for the helical cases, the success rate increased from 50% without restraints to 65% with the new dihedral angle restraints. This is also reflected in an overall improvement of the interface-RMSD (i-RMSD) by ∼1 Å and ∼10% increase in the total number of acceptable models.

### How Successful is Conformational Selection?

Despite the demonstrated performance improvement, how successful is conformation selection in recovering the proper peptide conformation for a given complex? During rigid-body docking, 6000 models are written to disk. Each model is effectively the results of 10 minimization trials (five, with for each automatic sampling of the 180° rotated solution), starting from one of the three conformations. Each conformation is thus represented equally in the total pool of models and it is up to scoring to selected the relevant models since the top 400 only is further refined.

**Figure 3:**
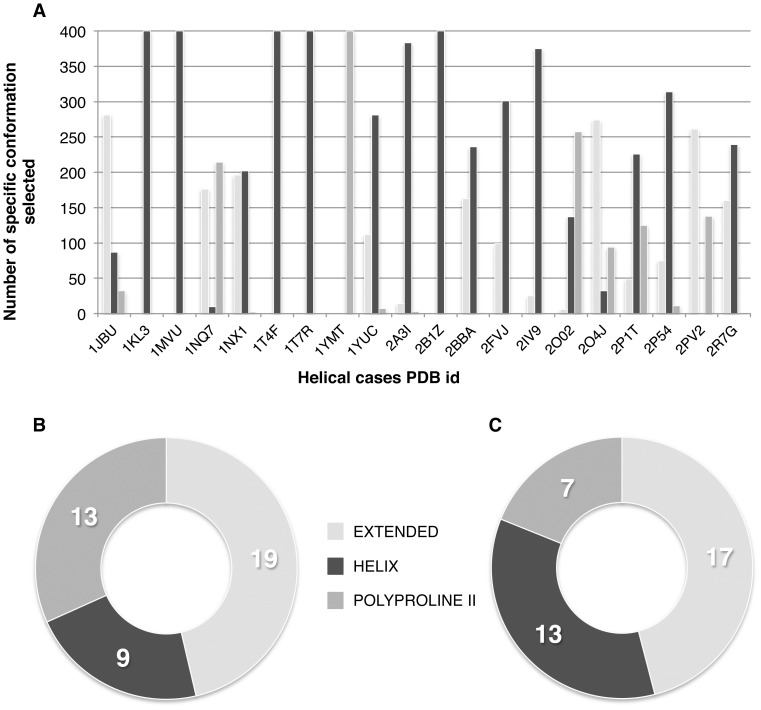
Performance of the conformational selection at the rigid-body stage of HADDOCK. The top 400 models are selected from the 6000 models generated based on their HADDOCK score. (**A**) Selection details for the 19 helical peptide cases. (**B**) Fractions of extended cases (41) with a predominant selection (i.e. majority of the selected conformations) coming from either extended, helical or polyproline II peptides. (**C**) Fraction of other cases (37) with a predominant selection coming from either extended, helical or polyproline II peptides.

The HADDOCK score at the rigid body stage is a combination of restraint, van der Waals, electrostatic and desolvation energies, together with a buried surface area term (HADDOCK_rigid_-score = 0.01 E_rest_ +0.01 E_vdw_ +1.0 E_elec_ +1.0 E_desol_ –0.01 BSA) [26], [27]. We assessed the pertinence of our scoring scheme for selecting the best conformation after it0 with respect to the peptide conformation in the complex. For the 19 helical cases, HADDOCK selected a majority of models coming from a starting helical conformation (**Figure 3A**) with 60% of the top 400 ranked models being in a helical conformation (corresponding to an enrichment factor of 1.80). The HADDOCK score performs also well for extended peptides since a majority of the selected models (∼50%) are generated from an ideal extended conformation of the peptide (**Figure 3B**) with 46% for the top 400 ranked models being in an extended conformation (corresponding to an enrichment factor of 1.32). Unsurprisingly, for cases with a disordered conformation of the peptide in the crystal structure, all three conformations are homogeneously selected from our input ensemble, with a slightly smaller contribution of polyproline II conformations (**Figure 3C**). Note that we also investigated whether our scoring function at the rigid body stage could be optimized to improve the selection performance by trying to maximize the number of acceptable models in the top 400 as described by [48]. Since, no significant improvement could be found, the weights were kept at their default values, which also correspond to the defaults settings for protein-protein and protein-DNA docking. This has the advantage that the same scoring function can be used for various molecule types or mixtures thereof.

### Unbound/Unbound Docking – A Challenging Task

Having established an efficient protein-peptide docking protocol using the bound form of the receptor (bound/unbound docking), we put it to the test on the real case of unbound docking, i.e. starting from the unbound form of the receptor protein and three conformations of the peptide. The original PeptiDB benchmark contains 47 cases with available unbound structures. Further analysis allowed us to identify 15 additional unbound structures, resulting in a total of 62 unbound/unbound cases (12 of which with helical peptide conformations).

To assess the difficulty of the docking in the presence of the unbound receptor, we measured, next to the conformational changes of the peptide itself (see above), the conformational changes occurring between the bound and the unbound forms of each protein at the interface. Eight out of the 62 unbound structures undergo conformational changes upon binding larger than 2.0 Å (**Table B in File S1**), with a maximum of 11.5 Å. Such conformational changes might be large enough to theoretically increase i-RMSD values above our acceptable limit even when the peptide is perfectly modeled onto the protein.

**Figure 4:**
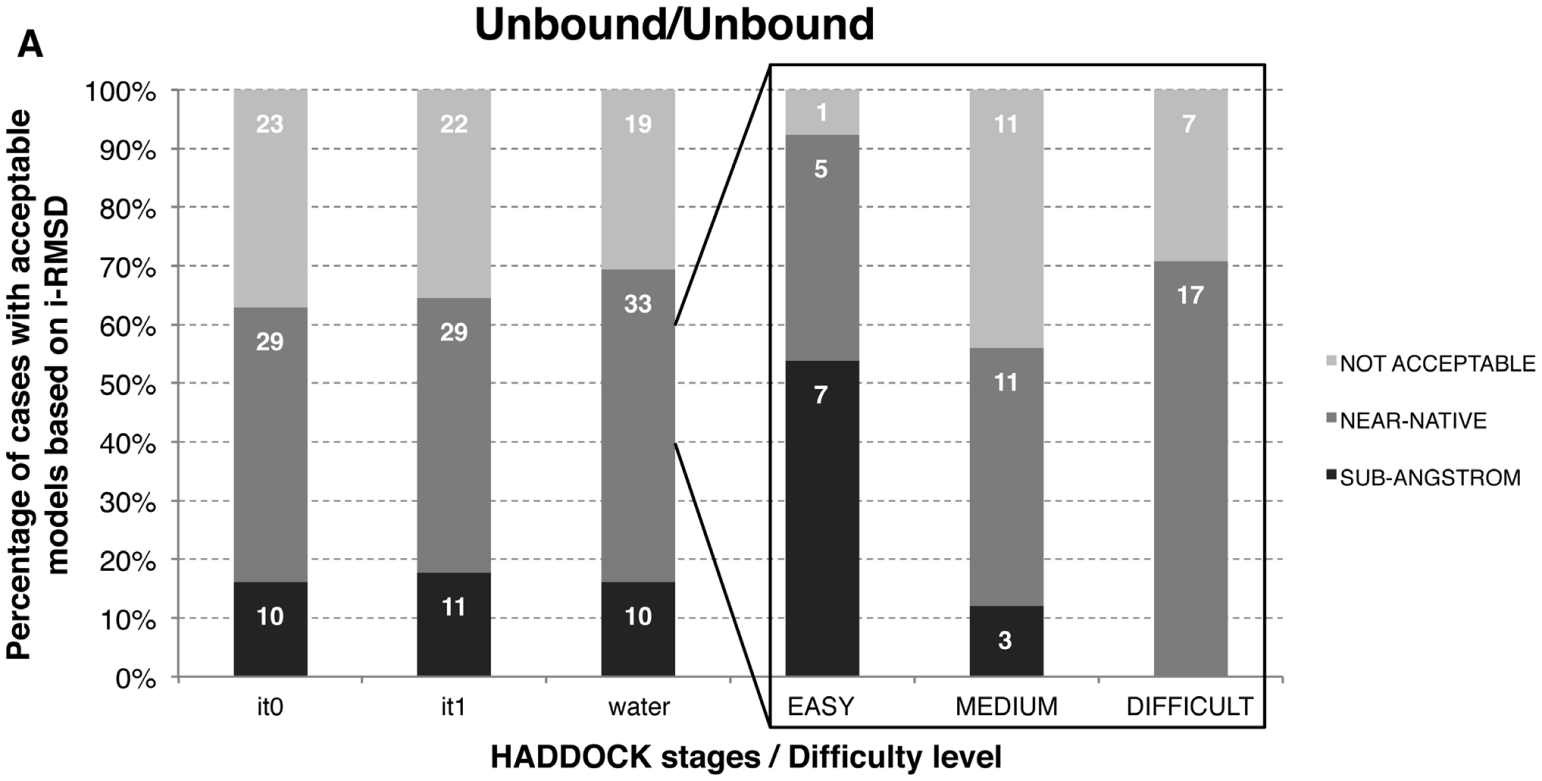
Unbound/unbound docking performance using the conformational selection/induced fit HADDOCK protocol. The percentages of near-native and sub-angstrom resolution models (see Methods) at the various stages (rigid-body (it0), semi-flexible (it1) and water refinement (water)) are reported in the left panels and were calculated over the 400 final models generated by HADDOCK. The right panels show the percentages after water refinement as a function of the docking difficulty.

Applying our conformational selection/induced fit docking protocol led to an impressive overall unbound/unbound docking success rate of 69.4%, meaning that 43 out of 62 cases presented acceptable models in the final stage of HADDOCK (**Figure 4**). Among the 19 cases that failed, eight complexes correspond to cases where the protein undergoes conformational changes above 2.0 Å and four to cases with conformational changes between 1.5 and 2.0 Å. We can also assess the quality of the modeling for the peptide independently from changes on the protein, by calculating the RMSD on the interface residues of the peptide after fitting on the interface of the protein receptor (ligand interface RMSD; l-i-RMSD). Using this measure, we got acceptable models for 65% of the cases when considering a 2.0 Å threshold for near-native solutions. This increases to 83% for a 2.5 Å threshold (**Figure A in File S1**).

### Determinants for Success: Ranking and Clustering

**Figure 5:**
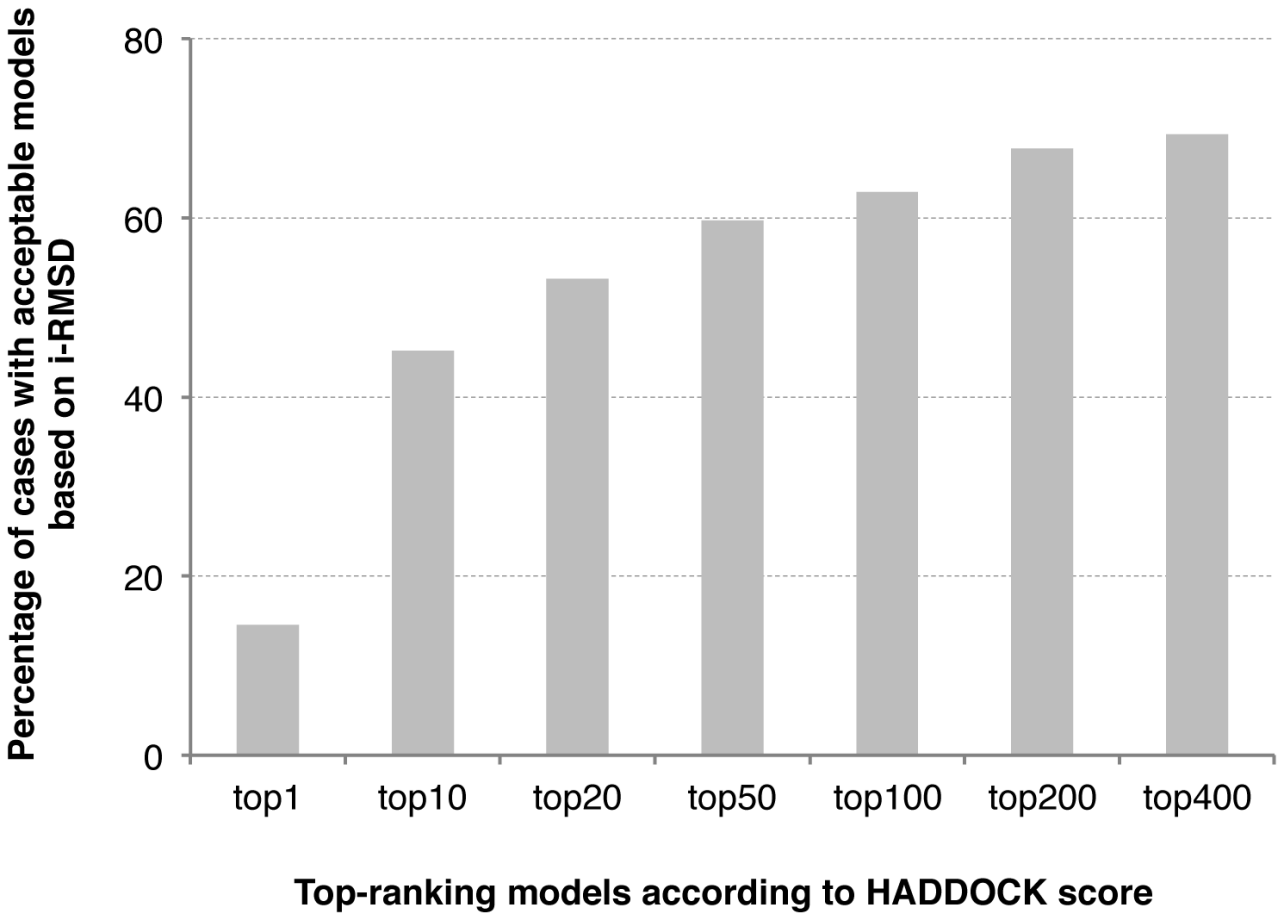
Success rate of unbound/unbound docking as a function of the number of top models considered. A docking is defined as successful it at least one near-native model is present within the topXX selected models.

HADDOCKs’ performance is very promising with acceptable models in the top 400 for ∼70% of the cases in unbound/unbound docking. How well are those models however scoring? To evaluate this we assessed the ranking performance of HADDOCK as a function of the top-scored N models (N ranging from 1 to 400) (**Figure 5**). This analysis reveals that we only reach 50% success rate when taking into consideration the top 20 models considering all cases. This underlines the difficulty of scoring consistently protein-peptide predictions as reported by a previous study [49]. On the other hand, the success rate only improves by ∼10% when going from top 50 to top 400, indicating that our scoring function can still reasonably well discriminate the ‘true negative’ (inaccurate models that have a lower score).

**Figure 6:**
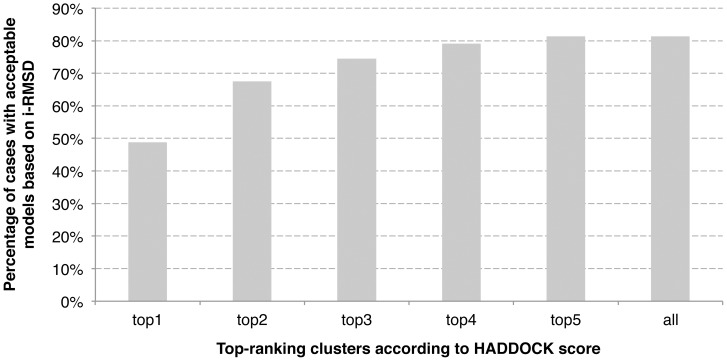
Clustering performance of HADDOCK in unbound/unbound docking onto acceptable cases (with at least one acceptable model) as a function of the number of clusters considered. A cluster is considered near-native if one of its top four member is of near-native quality or better.

**Figure 7:**
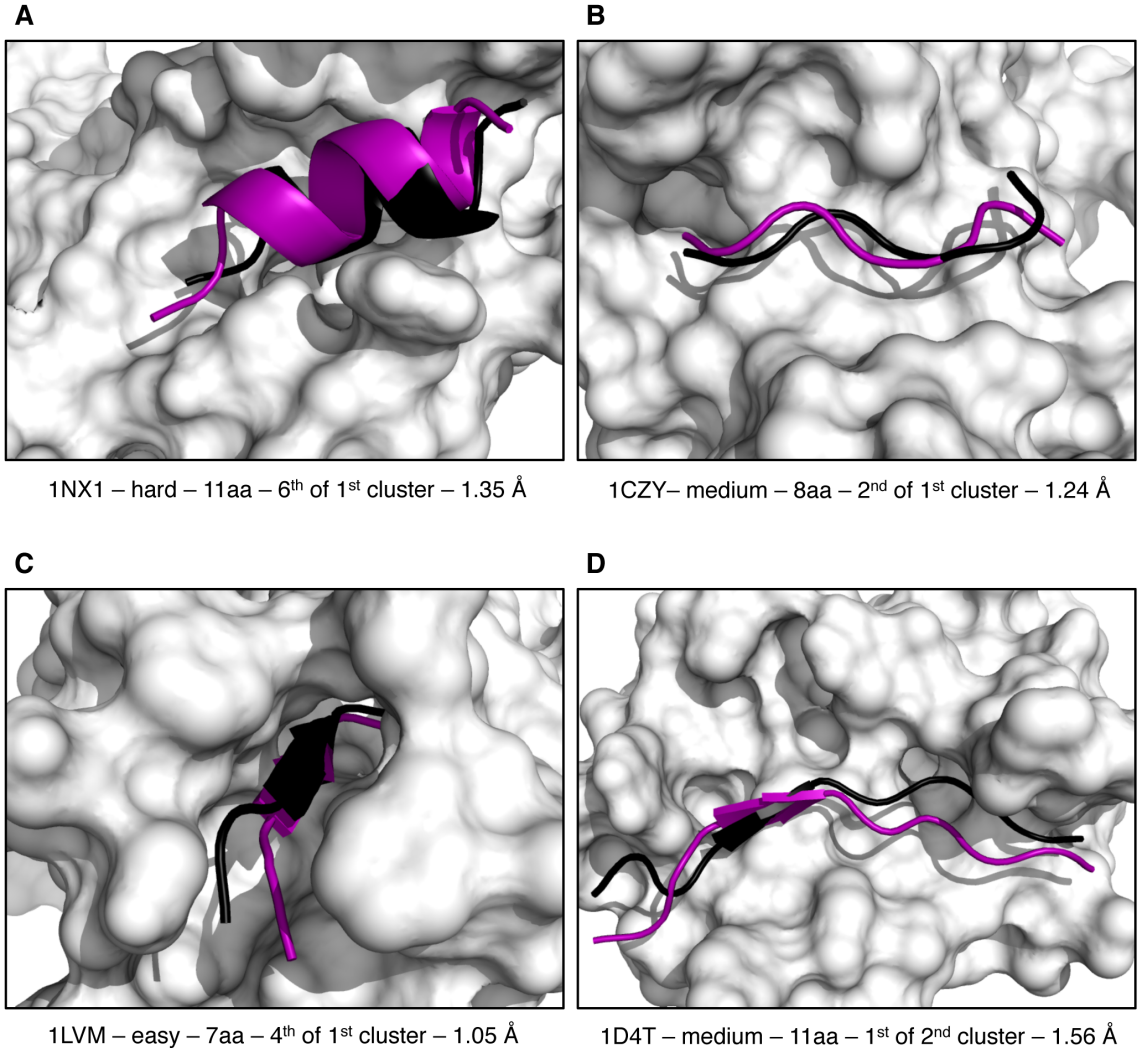
Examples of HADDOCK best models for the challenging unbound/unbound cases. The PDB-id as well as difficulty, peptide length, rank and i-RMSD values are indicated for each case. The model selected for illustration is the acceptable model with the best rank at the end of the HADDOCK process. The model peptide is shown in purple together with the reference peptide in the crystal structure of the complex in black. Docking model and crystal structure were superimposed on backbone atoms of the protein. The protein (crystal structure) is shown in surface representation. (A) 1NX1**,** (B) 1CZY**,** (C) 1LVM and (D) 1D4T. Figure generated with PyMol [56].

Ranking of individual models is however not the standard scoring procedure in HADDOCK: scoring is usually performed after clustering of the solutions and the final scoring is calculated on a per-cluster basis as the average score of the top 4 ranking models of each cluster. This has the advantage of smoothening the rather noisy contribution of individual energy terms, and in particular the electrostatic energy. Cluster-based ranking successfully ranks a near-native cluster at the top in ∼50% of the successful cases (cases for which HADDOCK generated at least one acceptable model in the top 400), and this quickly reaches 75% if the top three clusters are considered (**Figure 6**). For comparison, single structure scoring only ranks an acceptable model at the top of 21% of the cases (44% if we consider the top 3 ranking models) for which at least one acceptable model was generated. A few examples of unbound/unbound docking models obtained for challenging cases are illustrated in **Figure 7**.

## Discussion

Through this study, we developed a specific protocol for the flexible docking of short peptides (5−15 amino acids) onto proteins using HADDOCK. The protocol starts from an ensemble of three different conformations for the peptide, inspired by the conformational selection mechanism. This canonical ensemble does not aim at reproducing the free state conformations sampled by the peptide, but rather represents conformations often observed in protein-peptide complexes. Out of these, the favorable conformations selected at the rigid body stage are then subjected to an enhanced fully flexible refinement onto the protein, following the concept of induced fit. This approach enables HADDOCK to generate near native models for ∼80% of the cases for bound/unbound docking and ∼70% of the cases for unbound/unbound docking, and this, using the largest benchmark assembled to date. Furthermore, the HADDOCK cluster-based scoring scheme is shown to be efficient in retrieving an acceptable structure among the top three clusters in 75% of the successfully predicted cases, both for bound/unbound and unbound/unbound benchmarks. This represents quite a solid performance, especially considering that our method reaches an overall success rate of 93% (**Figure B in File S1)** when applied to the bound/bound dataset, which represents the ideal case and thus defines the upper limit of achievable success rate. Only seven cases among 101 were out of reach for HADDOCK because the peptides were deeply buried in the protein in the crystal structure. A drop of only 13.6% in success rate is observed from bound/bound to bound/unbound when we apply our ensemble-based flexible docking approach, and this is 23.6% for unbound/unbound. The performance of HADDOCK is however less impressive when considering the proportion of cases with sub-angstrom resolution models: it drops from 75% for bound/bound to 25% for bound/unbound docking, reaching only 17% in the case of unbound/unbound docking. This is still acceptable considering the difficulty of the problem. Together, these results confirm the relevance of our unified conformational selection/induced fit approach to the prediction of the 3D structure of protein-peptide complexes.

To further analyze our performance, we distinguished three levels of difficulties (easy/medium/difficult) for the docking based on the deviation between the bound form of the peptide and an ideal extended conformation (**Figure 1A**). This classification gives a direct indication about the amplitude of the conformational change required to fit the peptide at the interface, starting from an extended conformation. Interestingly, HADDOCK is not only able to provide reliable models for the easiest category of cases, but achieves also high success rates for the medium and difficult categories. Indeed, for the bound/unbound benchmark acceptable models are obtained in 87%, 79% and 76% of the easy, medium and difficult cases, respectively (**Figure 2C** **– right panel**). These percentages become really surprising for the unbound/unbound docking, with 92%, 57% and 71% of success rate for the respective easy, medium and difficult categories. This indicates that our protocol is not limited to the “easy” cases where the peptide binds as a stretched conformation (for example on SH3 domain) but can deal with more challenging cases as well.

**Figure 8:**
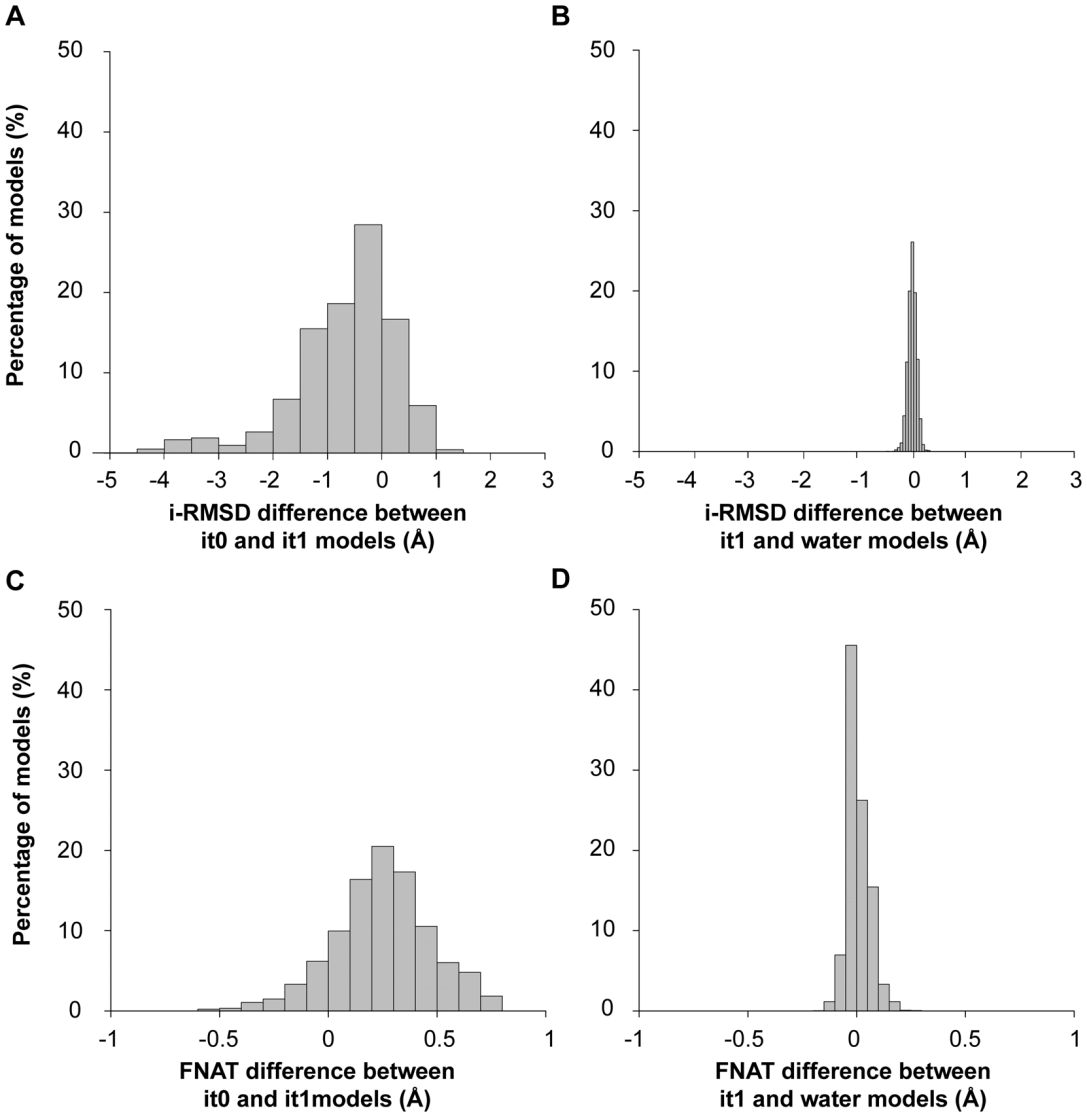
Difference in interface-RMSD (i-RMSD) and fraction of native contacts (Fnat) between models from various stages of HADDOCK (it0/it1/water) for unbound/unbound docking using our 3 conformation/enhanced flexibility protocol. The distributions are calculated from all generated models of the unbound/unbound docking benchmark. A negative i-RMSD difference value reflects an improvement (move toward the bound form) while a positive value indicates a deterioration of this i-RMSD. For Fnat this is reverse: a positive difference indicates an improvement. The impact of flexible refinement in torsion angle space (differences between rigid-body docking and flexible refinement (it1–it0)) is shown in **A)** i-RMSD diff and **C)** Fnat diff, and the impact of final water refinement (differences between flexible and water refinement (water-it1) is shown in **B)** i-RMSD diff and **D)** Fnat diff.

We analyzed the impact of the flexible refinement stages of HADDOCK on the quality of the generated acceptable models after water refinement, considering all unbound/unbound benchmark cases. The interface-RMSD improves by 0.7 Å on average during the semi-flexible refinement (it1), and by up to 5.0 Å for some cases (**Figure 8A**), while the change is only moderate (0.02 Å on average with a maximum of 0.42 Å) during the water refinement of HADDOCK (**Figure 8B**). The fraction of native contacts improves by 0.25 on average during it1, and by up to 0.79 in some cases (**Figure 8C**). Some substantial improvement is still observed during the water refinement for some models with a maximum of 0.28 (**Figure 8D**). All together, this shows that flexibility of the system is mostly handled during the flexible refinement stage (it1) of HADDOCK, while water refinement has the most impact on the fraction of native contacts and on the energetics.

**Figure 9:**
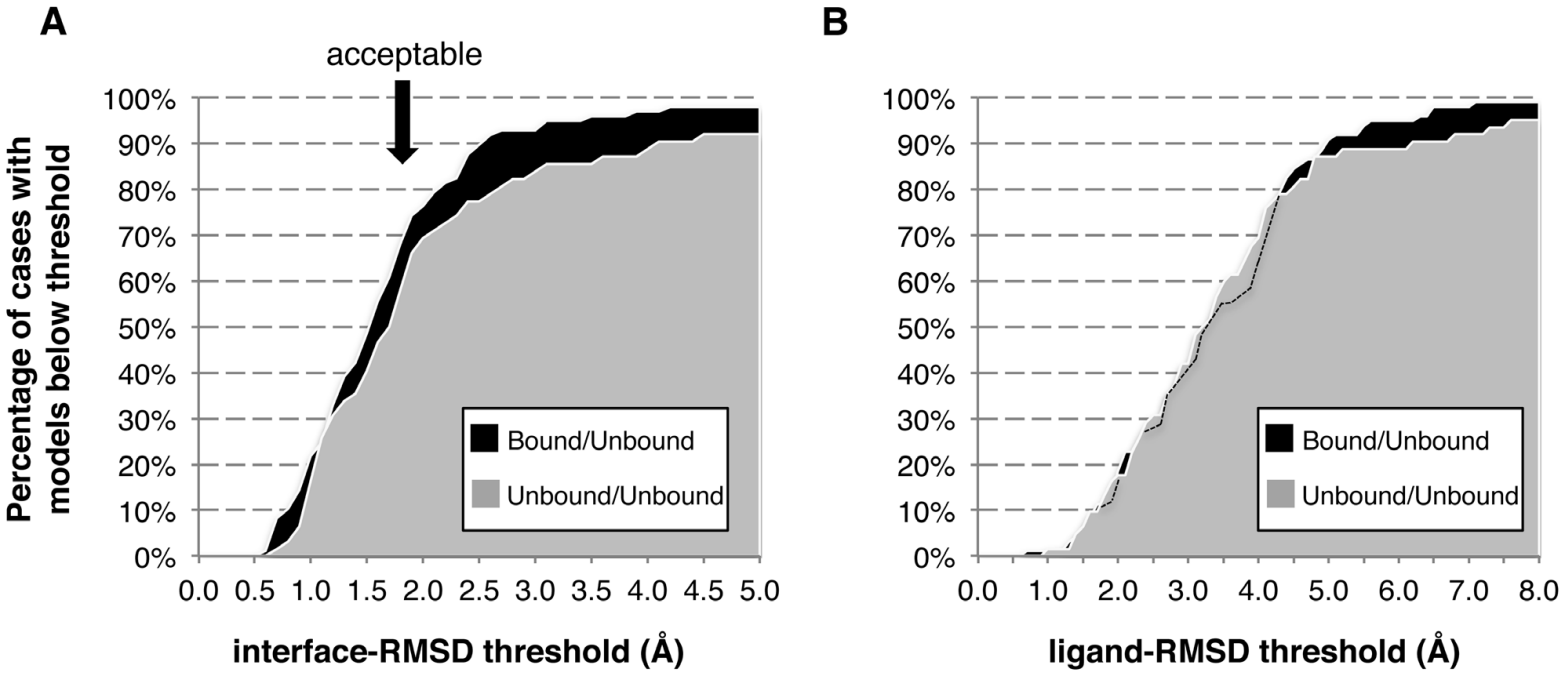
Impact of the (A) i-RMSD and (B) l-RMSD cutoffs defining a near-native solution on the docking performance. In this analysis, a docking run is defined as successful if at least one near-native model (for the selected cutoff) is generated within the pool of 400 water-refined models. Results are presented for both bound/unbound (97, black) and unbound/unbound (62, gray) cases.

We finally analyzed the impact of the chosen metric and associated cutoff on the success rate, for both interface-RMSD (i-RMSD, **Figure 9A**) and ligand-RMSD (l-RMSD, **Figure 9B**), the latter calculated on the entire peptide backbone. Interestingly, for i-RMSD cutoffs below 1.8 Å we observe that success rates remains similar for both bound/unbound and unbound/unbound docking (**Figure 9A**). Increasing the acceptability threshold above 2 Å results in more significant differences (∼10−15% in success rate) between bound/unbound and unbound/unbound benchmarks. The same analysis for l-RMSD reveals almost identical success rates for both bound/unbound and unbound/unbound benchmarks for thresholds below 5 Å (**Figure 9B**). The differences in success rates between bound/unbound and unbound/unbound docking based on various i-RMSD thresholds (which is not observed for l-RMSD thresholds) suggests that our performance for unbound/unbound docking is affected by the conformational changes of the protein when we discuss them with the CAPRI standard interface-RMSD definition. However, the results are much less influenced by the flexibility of the protein when only ligand or ligand-interface RMSD are considered. Ligand-RMSD based metrics alone therefore overestimate the performance of protein-peptide prediction algorithms. A proper assessment should not only measure the quality of the structural refinement of the peptides, but also address the flexibility of the protein receptor.

### Comparison with FlexPepDock and Dynadock

**Figure 10:**
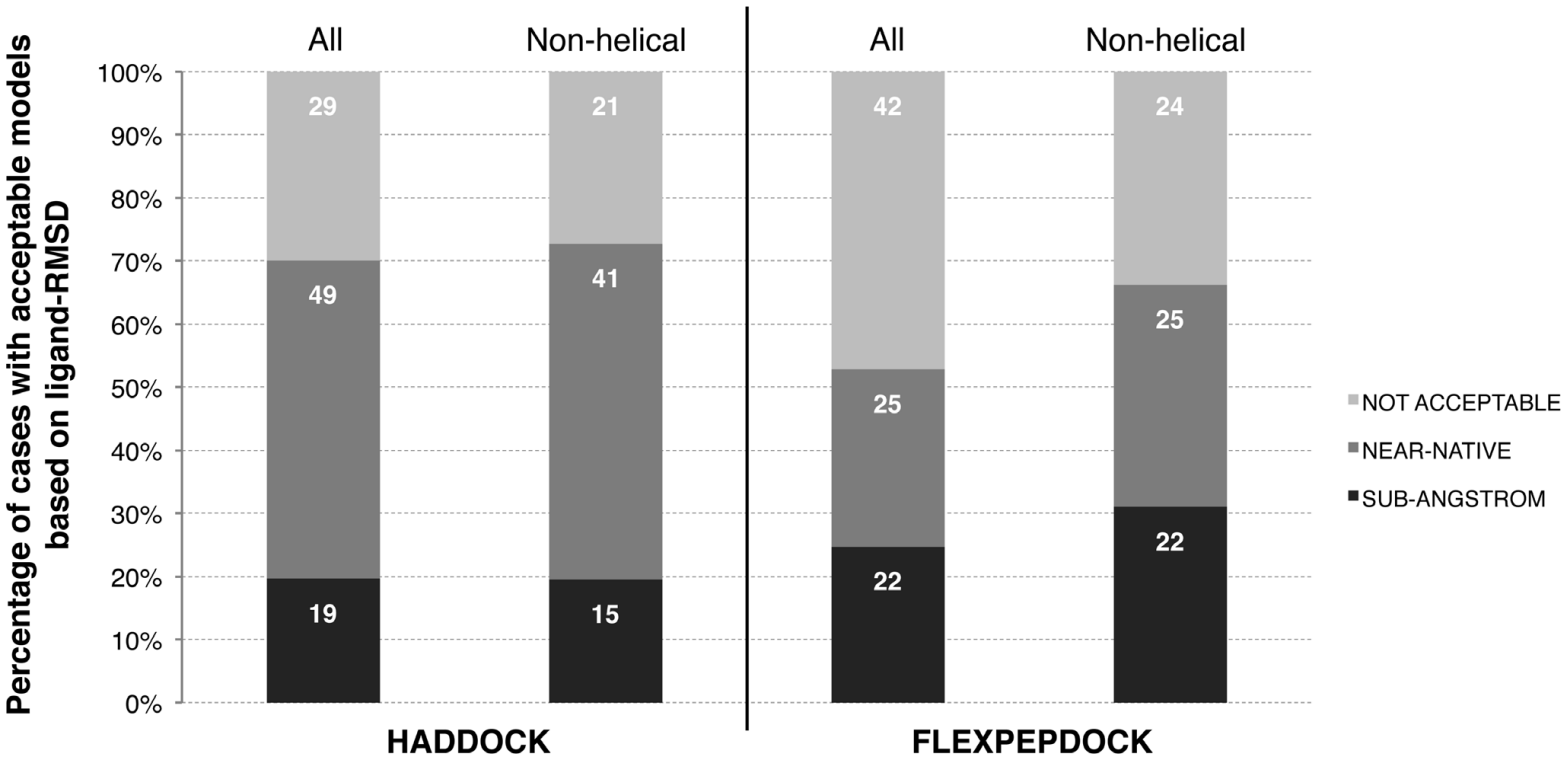
Comparison of the performance of HADDOCK and FlexPepDock. Percentage of cases with sub-angstrom and near-native models quality assessed by ligand-interface RMSD. The results are given for the whole bound/unbound benchmark and the ‘non-helical’ subset, as reported by FlexPepDock.

We compared the performance of our protocols with that of FlexPepDock, the only method that has been applied so far on the same protein-peptide dataset, concentrating on the bound/unbound benchmark since FlexPepDock results over the unbound/unbound dataset are not available. Both assume knowledge of the binding site: while HADDOCK uses a broad definition of the surface of interaction on the protein receptor, FlexPepDock assumes the knowledge of an anchoring residue at the interface. The two methods have significant differences in their approach of protein-peptide modeling but both allow a highly flexible sampling of the peptide. FlexPepDock’s performance, assessed on the RMSDs of the peptide backbone only, reaches an overall 52% success rate whereas HADDOCK was able to model acceptable structures for ∼70% of the cases using the same metric (**Figure 10**). Noticeably, FlexPepDock was not able to correctly model any helical cases. When restricted to the non-helical subset of their dataset, FlexPepDock reaches a success rate of 66%, with 49% of the cases containing at least one acceptable structure in the top five solutions. We repeated the same analysis for our results and we observed a similar success rate ∼73%, 53% of the cases with acceptable structures in the top five solutions. After clustering, however, HADDOCK reaches a similar success rate with 52% success rate considering the top three clusters. We should however note that FlexPepDock does generate more sub-angstrom resolution models, which might be explained by the different information used to guide the modeling (ambiguous interface versus anchoring residue).

Finally, we compared our protocol to the Dynadock method [49] that uses molecular dynamics simulations to account for the flexibility of the protein receptor. This method was benchmarked over 15 complexes that overlapped with our dataset. Dynadock defines a broad interface on the protein side (using a 6.5 Å threshold based on the crystal structure) and the peptides are initially “randomly” placed at their binding site, yet their orientation along the binding groove is restrained during the initial sampling. Using a ligand-interface RMSD with a threshold of 2.1 Å, Dynadock gets a final acceptable solution ranked as first for 11/15 cases. The same analysis for HADDOCK models gives acceptable solutions for 13/15 cases, including sub-angstrom solutions for six cases.

### Perspectives

Predicting large conformational changes remains a challenge as indicated by our failure to accurately predict cases where the protein undergoes large conformational changes upon binding. Among the eight cases with conformational changes at the receptor interface above 2.0 Å, two of them reveal a complete shift of one helix that ‘wraps’ the peptide, and the six others exhibit mostly local loop variations or secondary structure rearrangements. For the two first cases, a multi-body docking approach where the flexible domain of the protein would be considered as a separate body for the docking process could significantly improve the modeling. This was successfully applied in the past to protein-protein docking [50]. The problem is rather that such changes are difficult if not impossible to predict. The others cases could benefit from an initial refinement of the protein receptor alone, by molecular dynamics simulation for instance.

HADDOCK is a data-driven method that incorporates information during the docking process to narrow the search. In this work, we defined a broad binding site on the protein receptor directly from the crystal structures, which represents a best-case scenario. When no experimental information is available, one could rely on bioinformatics predictions or other computational methods to predict the interaction surfaces. Peptides seem indeed to recognize “hot spot” residues on the protein [51] that might be predictable. A number of approaches have been reported to predict peptide-binding site on proteins [52], [53]. Such predictors could be useful in the context of HADDOCK. Their performance for docking purposes will however have to be benchmarked in the future.

## Materials and Methods

### Protein-peptide Docking Benchmark

We used as benchmark the PeptiDB non-redundant dataset (sequence identity <70% for any two receptors) of 103 high-resolution (X-ray structures; <2 Å resolution) complexes of proteins bound to short peptides (5−15 amino acids long) [46]. This dataset also contains 47 high quality structures of unbound protein receptors that we complemented with 15 recently released unbound structures of the proteins, making together an unbound dataset of 62 cases (**Table C in File S1**).

Two complexes were removed from this dataset because of the total inaccessibility of the peptides binding site in the crystal structure (1XOC and 2D5W). For bound/unbound docking, four more cases were removed (1D4T, 1GYB, 2FMF and 2VJ0) because of problems with the coordinates of the proteins.

### Benchmark Classification

We divided our benchmark in three classes (easy/medium/difficult) based on the backbone RMSD between the conformation of the peptide in the crystal structure and its ideal extended conformation.

- easy: RMSD_bound/extended_ ≤4 Å
- medium: 4 Å<RMSD_bound/extended_ ≤8 Å
- difficult:RMSD_bound/extended_ >8 Å

The secondary structure of the peptides was assigned with STRIDE [54]. STRIDE encounters some issues to assign short amino-acid sequences that do not show consistent torsion angles for a particular conformation. We therefore considered as extended peptides those for which at least 80% of the sequence was in an ideal extended conformation. An ideal extended poly-alanine shows an intramolecular distance between two consecutive C_α_ equal to 3.46 Å. If the average C_α_–C_α_ distance is larger than 3.46×0.8 = 2.8 Å, then the peptide was considered as extended. This can be expressed mathematically in the following manner:
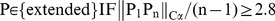
(1)where P indicates the peptide, 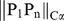 the end-to-end distance between the first and the last C_α_ and n the length of the peptide. Peptides were classified as helices when STRIDE [54] assigned more than half of their sequence in a helical conformation. All peptides that did not fall into the helical or extended classes were considered as disordered.

This classification scheme resulted in 21 helices, 42 extended and 38 disordered peptides.

To evaluate the conformational change between the unbound and bound forms of a protein, we calculated the RMSD of the protein interface, defining as interacting residues those within 10 Å from the peptide in the crystal structure of the complex (**Table B in File S1**).

### Solvent Accessibility Calculations

Solvent accessibility and buried surface areas were calculated using NACCESS [55]. We defined a residue as solvent accessible if its side-chain or its backbone has a relative accessibility over 40%.

### Modeling of Peptides Starting Conformations

PyMOL [56] was used to generate the three distinct conformation for every peptide. According to standard Ramachandran plots, the helical conformations were modeled with phi and psi angles −57° and −47°, respectively. Polyproline II conformations were built with −78° for phi and 149° for psi. Finally, the extended conformations of the peptides were generated using −139° for phi and −135° for psi.

### HADDOCK Settings

The bound conformations of the protein-peptide complexes were downloaded from the PDB databank [57]. The interface of the protein was defined from the crystal structure as follows: active residues (the defined interface for docking) on the protein side were defined as those within 5 Å from the peptide chain. Peptide residues were treated as passive. Random removal of restraints was turned off. Within the HADDOCK process, active residues are enforced to be part of the interface as much as possible by applying ambiguous interaction restraints while passive residue can be part of the interface. HADDOCK will thus try to satisfy as much interactions to active residues.

A typical HADDOCK docking run involves three consecutive steps. First, the molecules are randomly oriented and a rigid body energy minimization is performed (*it0*). The top ranked models (here top 400) are then addressed to the semi-flexible simulated annealing stage performed in torsion angle space (*it1*). In this study, this stage has been turned into a fully flexible simulated annealing as described below. Finally, the structures obtained after the semi-flexible simulated annealing are refined in an explicit solvent layer to further improve their scoring (*water*).

From preliminary tests on a small representative set (20 complexes), an increase in the number of flexible refinement steps by a factor four was found to lead to better conformations. Accordingly, the default number of MD steps for the flexible refinement stage was increased from 500/500/1000/1000 for the four stages of the flexible refinement to 2000/2000/4000/4000. These settings were subsequently applied to the entire benchmark.

The peptides were defined as fully flexible, meaning that side-chain and backbone flexibility is implemented from the start of the refinement stage (it1). On the protein side, only residues that are part of the interface (determined on the fly during docking) are treated as flexible, first allowing only side-chain flexibility followed by both backbone and side-chain flexibility in the final simulated annealing stage of it1. Note that this protocol thus allows for flexibility in the protein, even when starting from the bound form. The RMSD clustering cutoff was decreased from 7.5 Å to 5.0 Å to take into consideration the smaller size of protein-peptide interfaces. Finally, we specified charged Cter and Nter when we had indication of naturally occurring peptides and uncharged termini when the peptide was a fragment of protein or capped in the crystal structure.

#### Bound/unbound (extended) docking runs

The number of models generated during the three main stages of HADDOCK (it0/it1/water) was increased to 2000/400/400.

#### Bound/unbound (3 conformations) docking runs

The number of models generated during the three main steps of HADDOCK (it0/it1/water) was increased to 6000/400/400. In that way, each conformation is sampled 2000 times in the rigid body stage.

#### Unbound/unbound docking runs

The only change compared to bound/unbound (3 conformation) docking protocol is that the structure of the protein receptor in its unbound form was used, as downloaded from the Protein Data Bank.

All HADDOCK runs were launched on the WeNMR grid version [58] of the HADDOCK server (http://haddock.science.uu.nl/enmr/services/HADDOCK/haddock.php) that makes use of the European Grid Infrastructure (EGI) computing resources. On average, each run took between five and six hours to complete on the grid.

### Quality Assessment Criteria

In order to assess the quality of models generated by HADDOCK we criteria as defined by the CAPRI experiment [34], [59]. These were however reduced compare to standard protein-protein docking to account for the small size of the peptides. The quality of docking models was assessed using the interface RMSD (i-RMSD) as follows:

- Not acceptable: i-RMSD >2 Å
- Near-native prediction: 1 Å ≤ i-RMSD ≤2 Å
- High-quality (sub-angstrom) prediction: i- RMSD ≤1****Å

The i-RMSD is calculated on the backbone atoms of both protein and peptide residues that are within 10 Å of the partner molecules (as defined based on the crystal structure of the complex). The l-RMSD, when mentioned, is calculated on the backbone atoms of the peptide only, after fitting on the backbone atoms of the protein.

We further refer to as “acceptable models” any near-native or better (sub-angstrom) predictions.

## Supporting Information

File S1 **Table A** - Size of the protein interface measured in the different crystal structures (Native interface) and respective size of the surface used to define ambiguous restraints in HADDOCK to drive the modeling (Docking interface). Calculations have been done over the 97 cases successfully docked. **Table B** - Interface-RMSD between bound and unbound forms of the proteins and i-RMSD between the closest model generated by HADDOCK and the bound form of the protein for each case. **Table C** - List of protein-peptide complexes identifiers with their corresponding free forms when available. In red, the new entries added to PeptiDB in this study. **Figure A** **-** Success rate (% of benchmark cases with acceptable models) as a function of the ligand-interface RMSD cutoff. In this analysis, a docking run is defined as successful if at least one acceptable model (as defined by the l-i-RMSD cutoff) is generated among the 400 water-refined models. **Figure B -** Bound/bound docking performance using the default HADDOCK protocol. The percentages of near-native and sub-angstrom resolution models (see Methods) at the various stages (rigid-body - *it0*, semi-flexible - *it1* and water refinement - *water*) are reported in the left panels. The right panels show the percentages after water refinement as a function of the docking difficulty level.
(PDF)

## References

[1] Petsalaki E, Russell RB (2008) Peptide-mediated interactions in biological systems: new discoveries and applications. Curr Opin Biotechnol 19: 344–350. 18602004 10.1016/j.copbio.2008.06.004

[2] Bordner AJ, Abagyan R (2006) Ab initio prediction of peptide-MHC binding geometry for diverse class I MHC allotypes. Proteins 63: 512–526. 16470819 10.1002/prot.20831

[3] Hahn S, Kim D (2012) Transient protein-protein interaction of the SH3-peptide complex via closely located multiple binding sites. PLoS One 7: e32804. 22457720 10.1371/journal.pone.0032804 PMC3310816

[4] Lee HJ, Zheng JJ (2010) PDZ domains and their binding partners: structure, specificity, and modification. Cell Commun Signal 8: 8. 20509869 10.1186/1478-811X-8-8 PMC2891790

[5] Naider F, Anglister J (2009) Peptides in the treatment of AIDS. Curr Opin Struct Biol 19: 473–482. 19632107 10.1016/j.sbi.2009.07.003 PMC2763535

[6] Vaara M (2009) New approaches in peptide antibiotics. Curr Opin Pharmacol 9: 571–576. 19734091 10.1016/j.coph.2009.08.002

[7] Fjell CD, Hiss JA, Hancock REW, Schneider G (2012) Designing antimicrobial peptides: form follows function. Nature Reviews Drug Discovery 11: 37–51. 10.1038/nrd3591 22173434

[8] Wimley WC, Hristova K (2011) Antimicrobial peptides: successes, challenges and unanswered questions. J Membr Biol 239: 27–34. 21225255 10.1007/s00232-011-9343-0 PMC3166253

[9] Maes M, Loyter A, Friedler A (2012) Peptides that inhibit HIV-1 integrase by blocking its protein-protein interactions. FEBS J 279: 2795–2809. 22742518 10.1111/j.1742-4658.2012.08680.x

[10] Madine J, Doig AJ, Middleton DA (2008) Design of an N-methylated peptide inhibitor of alpha-synuclein aggregation guided by solid-state NMR. J Am Chem Soc 130: 7873–7881. 18510319 10.1021/ja075356q PMC2538558

[11] Yamin G, Ruchala P, Teplow DB (2009) A peptide hairpin inhibitor of amyloid beta-protein oligomerization and fibrillogenesis. Biochemistry 48: 11329–11331. 19877710 10.1021/bi901325g

[12] Frydman-Marom A, Shaltiel-Karyo R, Moshe S, Gazit E (2011) The generic amyloid formation inhibition effect of a designed small aromatic beta-breaking peptide. Amyloid 18: 119–127. 10.3109/13506129.2011.582902 21651439

[13] Svensen N, Walton JG, Bradley M (2012) Peptides for cell-selective drug delivery. Trends Pharmacol Sci 33: 186–192. 22424670 10.1016/j.tips.2012.02.002

[14] Shtatland T, Guettler D, Kossodo M, Pivovarov M, Weissleder R (2007) PepBank–a database of peptides based on sequence text mining and public peptide data sources. BMC Bioinformatics 8: 280. 17678535 10.1186/1471-2105-8-280 PMC1976427

[15] Vanhee P, Reumers J, Stricher F, Baeten L, Serrano L, et al (2009) PepX: a structural database of non-redundant protein-peptide complexes. Nucleic Acids Res 38: D545–551. 19880386 10.1093/nar/gkp893 PMC2808939

[16] Rubinstein M, Niv MY (2009) Peptidic modulators of protein-protein interactions: progress and challenges in computational design. Biopolymers 91: 505–513. 19226619 10.1002/bip.21164

[17] Cesareni G, Panni S, Nardelli G, Castagnoli L (2002) Can we infer peptide recognition specificity mediated by SH3 domains? FEBS Lett 513: 38–44. 11911878 10.1016/s0014-5793(01)03307-5

[18] Niv MY, Weinstein H (2005) A flexible docking procedure for the exploration of peptide binding selectivity to known structures and homology models of PDZ domains. J Am Chem Soc 127: 14072–14079. 16201829 10.1021/ja054195s

[19] Zhou Y, Abagyan R (1998) How and why phosphotyrosine-containing peptides bind to the SH2 and PTB domains. Fold Des 3: 513–522. 9889165 10.1016/S1359-0278(98)00067-4

[20] Stigler RD, Hoffmann B, Abagyan R, Schneider-Mergener J (1999) Soft docking an L and a D peptide to an anticholera toxin antibody using internal coordinate mechanics. Structure 7: 663–670. 10404595 10.1016/s0969-2126(99)80087-2

[21] Hetenyi C, van der Spoel D (2002) Efficient docking of peptides to proteins without prior knowledge of the binding site. Protein Sci 11: 1729–1737. 12070326 10.1110/ps.0202302 PMC2373668

[22] Dagliyan O, Proctor EA, D’Auria KM, Ding F, Dokholyan NV (2011) Structural and dynamic determinants of protein-peptide recognition. Structure 19: 1837–1845. 22153506 10.1016/j.str.2011.09.014 PMC3240807

[23] Raveh B, London N, Schueler-Furman O (2010) Sub-angstrom modeling of complexes between flexible peptides and globular proteins. Proteins 78: 2029–2040. 20455260 10.1002/prot.22716

[24] London N, Raveh B, Cohen E, Fathi G, Schueler-Furman O (2011) Rosetta FlexPepDock web server–high resolution modeling of peptide-protein interactions. Nucleic Acids Res 39: W249–253. 21622962 10.1093/nar/gkr431 PMC3125795

[25] Raveh B, London N, Zimmerman L, Schueler-Furman O (2011) Rosetta FlexPepDock ab-initio: simultaneous folding, docking and refinement of peptides onto their receptors. PLoS One 6: e18934. 21572516 10.1371/journal.pone.0018934 PMC3084719

[26] Dominguez C, Boelens R, Bonvin AM (2003) HADDOCK: a protein-protein docking approach based on biochemical or biophysical information. J Am Chem Soc 125: 1731–1737. 12580598 10.1021/ja026939x

[27] de Vries SJ, van Dijk AD, Krzeminski M, van Dijk M, Thureau A, et al (2007) HADDOCK versus HADDOCK: new features and performance of HADDOCK2.0 on the CAPRI targets. Proteins 69: 726–733. 17803234 10.1002/prot.21723

[28] Tzakos AG, Fuchs P, van Nuland NA, Troganis A, Tselios T, et al (2004) NMR and molecular dynamics studies of an autoimmune myelin basic protein peptide and its antagonist: structural implications for the MHC II (I-Au)-peptide complex from docking calculations. Eur J Biochem 271: 3399–3413. 15291817 10.1111/j.1432-1033.2004.04274.x

[29] Musi V, Birdsall B, Fernandez-Ballester G, Guerrini R, Salvatori S, et al (2006) New approaches to high-throughput structure characterization of SH3 complexes: the example of Myosin-3 and Myosin-5 SH3 domains from S. cerevisiae. Protein Sci 15: 795–807. 16600966 10.1110/ps.051785506 PMC2242487

[30] Gelis I, Bonvin AM, Keramisanou D, Koukaki M, Gouridis G, et al (2007) Structural basis for signal-sequence recognition by the translocase motor SecA as determined by NMR. Cell 131: 756–769. 18022369 10.1016/j.cell.2007.09.039 PMC2170882

[31] Schneider T, Kruse T, Wimmer R, Wiedemann I, Sass V, et al (2010) Plectasin, a fungal defensin, targets the bacterial cell wall precursor Lipid II. Science 328: 1168–1172. 20508130 10.1126/science.1185723

[32] Casares S, Ab E, Eshuis H, Lopez-Mayorga O, van Nuland NA, et al (2007) The high-resolution NMR structure of the R21A Spc-SH3: P41 complex: understanding the determinants of binding affinity by comparison with Abl-SH3. BMC Struct Biol 7: 22. 17407569 10.1186/1472-6807-7-22 PMC1853097

[33] Karaca E, Melquiond AS, de Vries SJ, Kastritis PL, Bonvin AM (2010) Building macromolecular assemblies by information-driven docking: introducing the HADDOCK multibody docking server. Mol Cell Proteomics 9: 1784–1794. 20305088 10.1074/mcp.M000051-MCP201 PMC2938057

[34] Janin J, Henrick K, Moult J, Eyck LT, Sternberg MJ, et al (2003) CAPRI: a Critical Assessment of PRedicted Interactions. Proteins 52: 2–9. 12784359 10.1002/prot.10381

[35] Lensink MF, Wodak SJ (2010) Docking and scoring protein interactions: CAPRI 2009. Proteins 78: 3073–3084. 20806235 10.1002/prot.22818

[36] de Vries SJ, van Dijk M, Bonvin AM (2010) The HADDOCK web server for data-driven biomolecular docking. Nat Protoc 5: 883–897. 20431534 10.1038/nprot.2010.32

[37] Fischer E (1894) Einfluss der configuration auf die Wirkung der enzyme. Berichte der Deutchen Chemischen Gesellschaft 27: 2985–2993.

[38] Koshland DE Jr (1959) Enzyme flexibility and enzyme action. J Cell Comp Physiol 54: 245–258. 14411189 10.1002/jcp.1030540420

[39] Kumar S, Ma B, Tsai CJ, Sinha N, Nussinov R (2000) Folding and binding cascades: dynamic landscapes and population shifts. Protein Sci 9: 10–19. 10739242 10.1110/ps.9.1.10 PMC2144430

[40] Rubin MM, Changeux JP (1966) On the nature of allosteric transitions: implications of non-exclusive ligand binding. J Mol Biol 21: 265–274. 5972463 10.1016/0022-2836(66)90097-0

[41] Monod J, Wyman J, Changeux JP (1965) On the Nature of Allosteric Transitions: A Plausible Model. J Mol Biol 12: 88–118. 14343300 10.1016/s0022-2836(65)80285-6

[42] Hammes GG, Chang YC, Oas TG (2009) Conformational selection or induced fit: a flux description of reaction mechanism. Proc Natl Acad Sci U S A 106: 13737–13741. 19666553 10.1073/pnas.0907195106 PMC2728963

[43] Csermely P, Palotai R, Nussinov R (2010) Induced fit, conformational selection and independent dynamic segments: an extended view of binding events. Trends Biochem Sci 35: 539–546. 20541943 10.1016/j.tibs.2010.04.009 PMC3018770

[44] Changeux JP, Edelstein S (2011) Conformational selection or induced fit? 50 years of debate resolved. F1000 Biol Rep 3: 19. 21941598 10.3410/B3-19 PMC3169905

[45] Weikl TR, von Deuster C (2009) Selected-fit versus induced-fit protein binding: kinetic differences and mutational analysis. Proteins 75: 104–110. 18798570 10.1002/prot.22223

[46] London N, Movshovitz-Attias D, Schueler-Furman O (2010) The structural basis of peptide-protein binding strategies. Structure 18: 188–199. 20159464 10.1016/j.str.2009.11.012

[47] Diella F, Haslam N, Chica C, Budd A, Michael S, et al (2008) Understanding eukaryotic linear motifs and their role in cell signaling and regulation. Front Biosci 13: 6580–6603. 18508681 10.2741/3175

[48] Pons C, D’Abramo M, Svergun DI, Orozco M, Bernado P, et al (2010) Structural characterization of protein-protein complexes by integrating computational docking with small-angle scattering data. J Mol Biol 403: 217–230. 20804770 10.1016/j.jmb.2010.08.029

[49] Antes I (2010) DynaDock: A new molecular dynamics-based algorithm for protein-peptide docking including receptor flexibility. Proteins 78: 1084–1104. 20017216 10.1002/prot.22629

[50] Karaca E, Bonvin AM (2011) A multidomain flexible docking approach to deal with large conformational changes in the modeling of biomolecular complexes. Structure 19: 555–565. 21481778 10.1016/j.str.2011.01.014

[51] Stein A, Aloy P (2008) Contextual specificity in peptide-mediated protein interactions. PLoS One 3: e2524. 18596940 10.1371/journal.pone.0002524 PMC2438476

[52] Trabuco LG, Lise S, Petsalaki E, Russell RB (2012) PepSite: prediction of peptide-binding sites from protein surfaces. Nucleic Acids Res 40: W423–427. 22600738 10.1093/nar/gks398 PMC3394340

[53] Ben-Shimon A, Eisenstein M (2010) Computational mapping of anchoring spots on protein surfaces. J Mol Biol 402: 259–277. 20643147 10.1016/j.jmb.2010.07.021

[54] Heinig M, Frishman D (2004) STRIDE: a web server for secondary structure assignment from known atomic coordinates of proteins. Nucleic Acids Res 32: W500–502. 15215436 10.1093/nar/gkh429 PMC441567

[55] Hubbard SJ, Thornton JM (1993) NACCESS. 2.1.1 ed. Manchester.

[56] Schrodinger LLC (2010) The PyMOL Molecular Graphics System, Version 1.3r1.

[57] Berman HM, Westbrook J, Feng Z, Gilliland G, Bhat TN, et al (2000) The Protein Data Bank. Nucleic Acids Res 28: 235–242. 10592235 10.1093/nar/28.1.235 PMC102472

[58] Wassenaar TA, van Dijk M, Loureiro-Ferreira N, van der Schot G, de Vries SJ, et al.. (2011) WeNMR: structural biology on the grid. CEUR. [1]–[8] %@ 1613–0073.

[59] Mendez R, Leplae R, De Maria L, Wodak SJ (2003) Assessment of blind predictions of protein-protein interactions: current status of docking methods. Proteins 52: 51–67. 12784368 10.1002/prot.10393

