# Supplementary material for: A Unified Conformational Selection and Induced Fit Approach to Protein-Peptide Docking
Source: PLoS One. 2013 Mar 13;8(3):e58769. doi: 10.1371/journal.pone.0058769 (PMC3596317; doi:10.1371/journal.pone.0058769)

Supplementary Material

A unified conformational selection and induced fit approach to protein-peptide docking

Mikaël Trellet[1,2], Adrien SJ Melquiond[1], Alexandre MJJ Bonvin[1]

[1] *Computational Structural Biology group, Bijvoet Center for Biomolecular Research, Faculty of Science - Chemistry, Utrecht University, 3584 CH Utrecht, The Netherlands*

[2] Current address: *Groupe VENISE, CNRS LIMSI, 91403 Orsay CEDEX, France*

This supplementary material contains three tables and two figures.

Table A - Size of the protein interface measured in the different crystal structures (Native interface) and respective size of the surface used to define ambiguous restraints in HADDOCK to drive the modeling (Docking interface). Calculations have been done over the 97 cases successfully docked.

PDBid	Native interface ($Å^2$)	Docking interface ($Å^2$)
1AWR	357	747
1CE1	350	918
1CKA	432	1092
1CZY	426	1091
1DDV	225	635
1DKX	472	838
1EG4	449	1236
1ELW	454	1005
1ER8	684	1230
1H6W	857	2363
1HC9	598	1590
1I8K	503	1267
1IAK	973	2080
1IHJ	277	680
1JBU	875	2018
1JD5	398	878
1JWG	391	1152
1KL3	373	829
1KLU	938	1967
1LVM	539	1728
1MFG	419	1114
1MVU	423	808
1N12	861	2294
1N7F	440	940
1NLN	735	1621
1NQ7	539	1107
1NTV	593	1531
1NVR	300	779
1NX1	476	1274
1OAI	344	928
1OU8	449	1193
1PZ5	516	1164
1QKZ	472	1154
1RXZ	581	1709
1SE0	376	944
1SFI	615	1215
1SSH	416	1143
1SVZ	471	1150
1T4F	463	1070
1T7R	463	953
1TP5	411	1009
1TW6	338	877
1U00	595	1234
1U8I	450	1077
1U9L	622	1185
1UJ0	381	1096
1VZQ	380	885
1W9E	413	741
1X2R	444	1040
1YMT	429	905
1YPH	511	1234
1YUC	569	1167
1YWO	379	943
1Z9O	673	1974
1ZUK	735	1495
2A3I	492	957
2AI4	540	1560
2AK5	365	754
2B1Z	477	768
2B9H	582	1312
2BBA	660	1319
2C3I	481	943
2CCH	610	1099
2D0N	375	894
2DS8	347	1184
2DZE	702	1574
2FGR	354	907
2FNT	596	1476
2FOJ	322	958
2FVJ	481	920
2H9M	340	535
2HO2	246	591
2HPL	290	803
2IPU	487	1037
2IV9	565	1258
2J6F	423	1069
2JAM	350	880
2O02	786	1872
2O4J	506	961
2O9V	358	759
2OTU	755	1494
2P0W	739	1379
2P1K	556	1541
2P1T	503	1313
2P54	571	1237
2PUY	483	1460
2PV2	978	2156
2QOS	680	1150
2R7G	755	1810
2V3S	324	835
2ZJD	584	1205
3BFQ	1008	2470
3BU3	907	2224
3BWA	624	1302
3CVP	417	781
3D1E	412	1238
3D9T	308	810
average	517	1207

Table B - Interface-RMSD between bound and unbound forms of the proteins and i-RMSD between the closest model generated by HADDOCK and the bound form of the protein for each case.

PDBid	i-RMSD bound vs unbound (Å)	i-RMSD bound vs best model (Å)
1AWR	0.30	0.77
1CE1	1.88	2.53
1CKA	1.48	1.94
1CZY	0.27	0.93
1D4T	1.60	1.56
1DDV	0.81	1.78
1EG4	0.94	9.53
1ER8	0.24	1.24
1GYB	0.15	5.04
1HC9	3.50	4.50
1JD5	4.15	5.84
1JWG	0.45	1.11
1KL3	1.59	2.61
1KLU	0.33	1.59
1LVM	0.47	0.93
1MFG	0.67	1.26
1N7F	0.40	0.91
1NQ7	0.98	1.05
1NVR	0.23	1.64
1NX1	0.93	1.08
1OAI	1.49	2.35
1OU8	0.61	1.54
1PZ5	0.32	1.44
1RXZ	1.47	1.79
1SE0	0.28	1.03
1SFI	0.24	2.75
1T4F	1.50	1.72
1T7R	0.41	1.10
1TP5	0.49	0.88
1TW6	0.13	0.64
1VZQ	1.77	2.27
1W9E	0.56	0.86
1X2R	0.26	1.37
1YWO	0.35	2.03
2A3I	0.29	1.74
2AK5	0.54	1.00
2B1Z	4.25	6.58
2B9H	0.32	1.85
2C3I	0.20	1.20
2CCH	0.24	1.84
2DS8	0.58	0.96
2FGR	0.28	1.84
2FNT	0.60	1.45
2FOJ	0.28	1.15
2FVJ	0.31	1.43
2H9M	2.09	4.09
2HO2	0.55	1.83
2HPL	0.23	1.10
2IPU	2.30	3.03
2J6F	0.29	0.96
2O02	0.55	1.60
2O4J	0.45	1.98
2O9V	0.31	1.85
2P1T	11.48	8.77
2P54	0.73	1.73
2PUY	1.06	3.00
2QOS	0.90	2.36
2R7G	1.20	1.62
2VJ0	3.41	3.98
2ZJD	1.11	2.11
3D1E	0.38	1.02
3D9T	2.71	3.55

Table C - List of protein-peptide complexes identifiers with their corresponding free forms when available. In red, the new entries added to PeptiDB in this study.

PDBid complex	PDBid free protein
1AWR	2ALF:A
1CE1	**1UM5**
1CKA	2DVJ:A
1CZY	1CZZ:C
1D4T	**1D1Z:C**
1DDV	1I2H:A
1DKX	-
1EG4	1EG3:A
1ELW	-
1ER8	1OEW:A
1GYB	1GY7:B
1H6W	-
1HC9	**2ABX:A**
1I8K	-
1IAK	-
1IHJ	-
1JBU	2BZ6:H
1JD5	1QBH:A
1JWG	**1JWF:A**
1KL3	2RTM:A
1KLU	**1PYW:AB**
1LVM	1LVB:B
1MFG	2H3L:A
1MVU	-
1N12	-
1N7F	1N7E:A
1NLN	-
1NQ7	1N83:A
1NTV	-
1NVR	2QHN:A
1NX1	1ALV:A
1OAI	1GO5:A
1OU8	1OU9:A
1PZ5	**1M7D:AB**
1QKZ	-
1RXZ	1RWZ:A
1SE0	**3SIQ :A**
1SFI	1UTN:A
1SSH	1OOT:A
1SVZ	-
1T4F	1Z1M:A
1T7R	2AM9:A
1TP5	**1TQ3:A**
1TW6	**2I3I :A**
1U00	-
1U8I	-
1U9L	-
1UJ0	-
1VZQ	1JWT:A
1W9E	1R6J:A
1X2R	1X2J:A
1XOC	-
1YMT	-
1YPH	-
1YUC	**3TX7:B**
1YWO	1Y-M:A
1Z9O	**1Z9L:A**
1ZUK	-
2A3I	2AA2:A
2AI4	-
2AK5	2G6F:X
2B1Z	**2IOG:A**
2B9H	2B9F:A
2BBA	-
2C3I	2J2I:B
2CCH	1H1R:B
2D0N	-
2D5W	-
2DS8	2DS7:A
2DZE	-
2FGR	2FGQ:X
2FMF	1JBE:A
2FNT	**3HAU**
2FOJ	2F1W:A
2FVJ	2HWQ:A
2H9M	2H14:A
2HO2	2E45:A
2HPL	2HPJ:A
2IPU	**1YEJ:HL**
2IV9	**1E42:AB**
2J6F	**2J6K:A**
2JAM	-
2O02	**3RDH:A**
2O4J	1IE9:A
2O9V	2O9S:A
2OTU	-
2P0W	-
2P1K	-
2P1T	**3NSQ:A**
2P54	1I7G:A
2PUY	**2YQL:A**
2PV2	-
2QOS	1LF7:A
2R7G	**3POM:A**
2V3S	-
2VJ0	1B9K:A
2ZJD	1V49:A
3BFQ	-
3BU3	**3EKK:A**
3BWA	-
3CVP	-
3D1E	3D1G:A
3D9T	1QBH:A

Figure A. Success rate (% of benchmark cases with acceptable models) as a function of the ligand-interface RMSD cutoff. In this analysis, a docking run is defined as successful if at least one acceptable model (as defined by the l-i-RMSD cutoff) is generated among the 400 water-refined models.

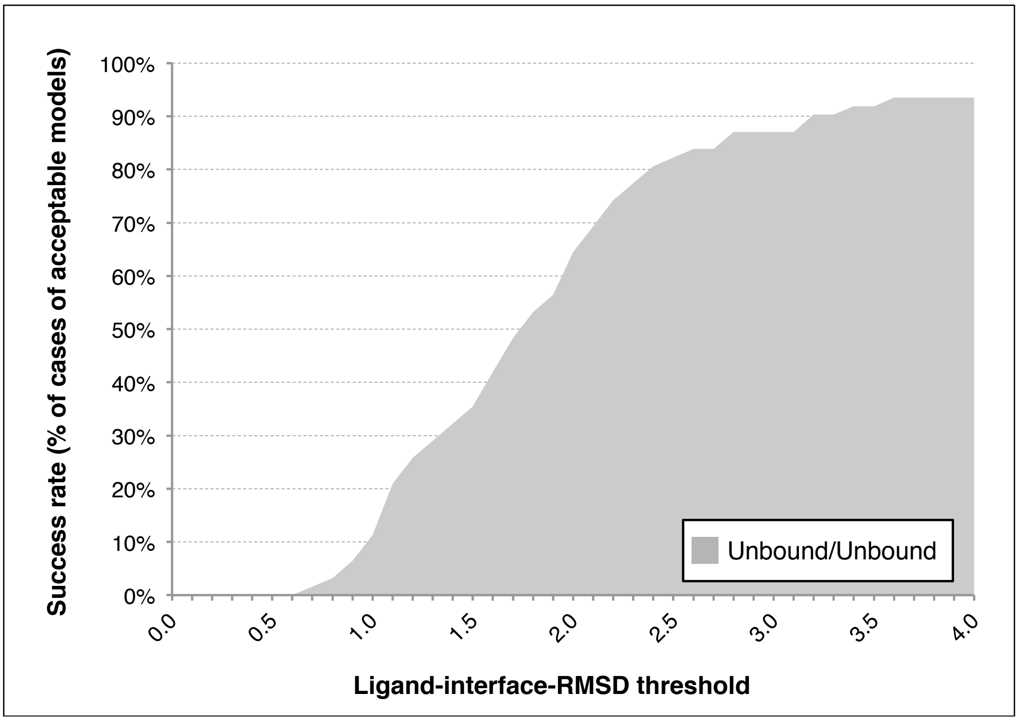

Figure B - Bound/bound docking performance using the default HADDOCK protocol. The percentages of near-native and sub-angstrom resolution models (see Methods) at the various stages (rigid-body - *it0*, semi-flexible - *it1* and water refinement - *water*) are reported in the left panels. The right panels show the percentages after water refinement as a function of the docking difficulty level.

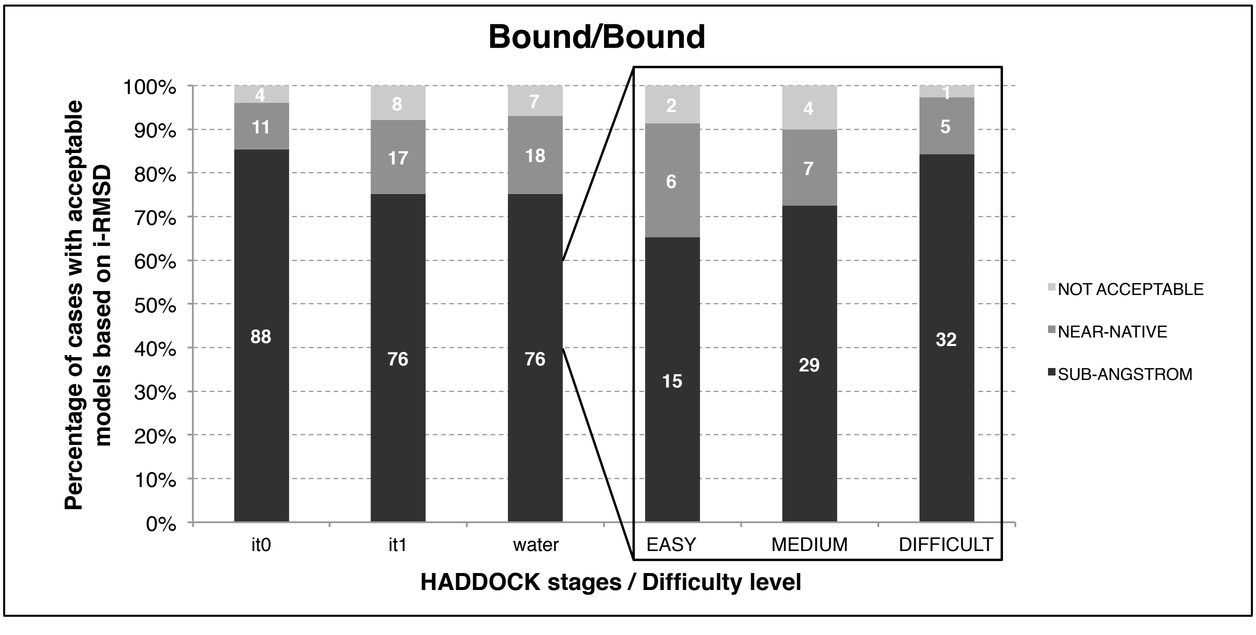

Supplement: File S1 — Table A - Size of the protein interface measured in the different crystal structures (Native interface) and respective size of the surface used to define ambiguous restraints in HADDOCK to drive the modeling (Docking interface). Calculations have been done over the 97 cases successfully docked. Table B - Interface-RMSD between bound and unbound forms of the proteins and i-RMSD between the closest model generated by HADDOCK and the bound form of the protein for each case. Table C - List of protein-peptide complexes identifiers with their corresponding free forms when available. In red, the new entries added to PeptiDB in this study. Figure A - Success rate (% of benchmark cases with acceptable models) as a function of the ligand-interface RMSD cutoff. In this analysis, a docking run is defined as successful if at least one acceptable model (as defined by the l-i-RMSD cutoff) is generated among the 400 water-refined models. Figure B - Bound/bound docking performance using the default HADDOCK protocol. The percentages of near-native and sub-angstrom resolution models (see Methods) at the various stages (rigid-body - it0, semi-flexible - it1 and water refinement - water) are reported in the left panels. The right panels show the percentages after water refinement as a function of the docking difficulty level. (PDF) [file pone.0058769.s001.pdf]
